# Supplementary figures and images for: The Infectious Dose Shapes Vibrio cholerae Within-Host Dynamics
Source: mSystems. 2021 Dec 7;6(6):e00659-21. doi: 10.1128/mSystems.00659-21 (PMC8651084; doi:10.1128/mSystems.00659-21)

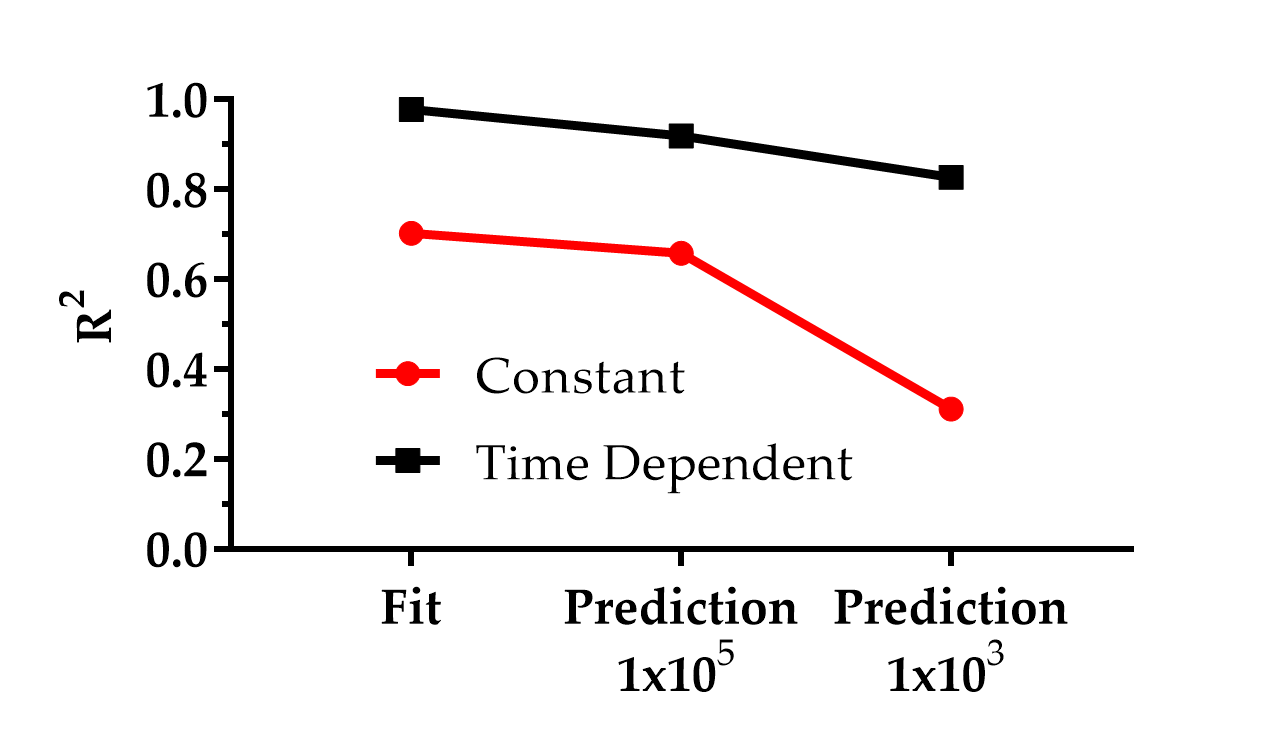

Supplement: FIG S1 [file msystems.00659-21-sf001.tif]

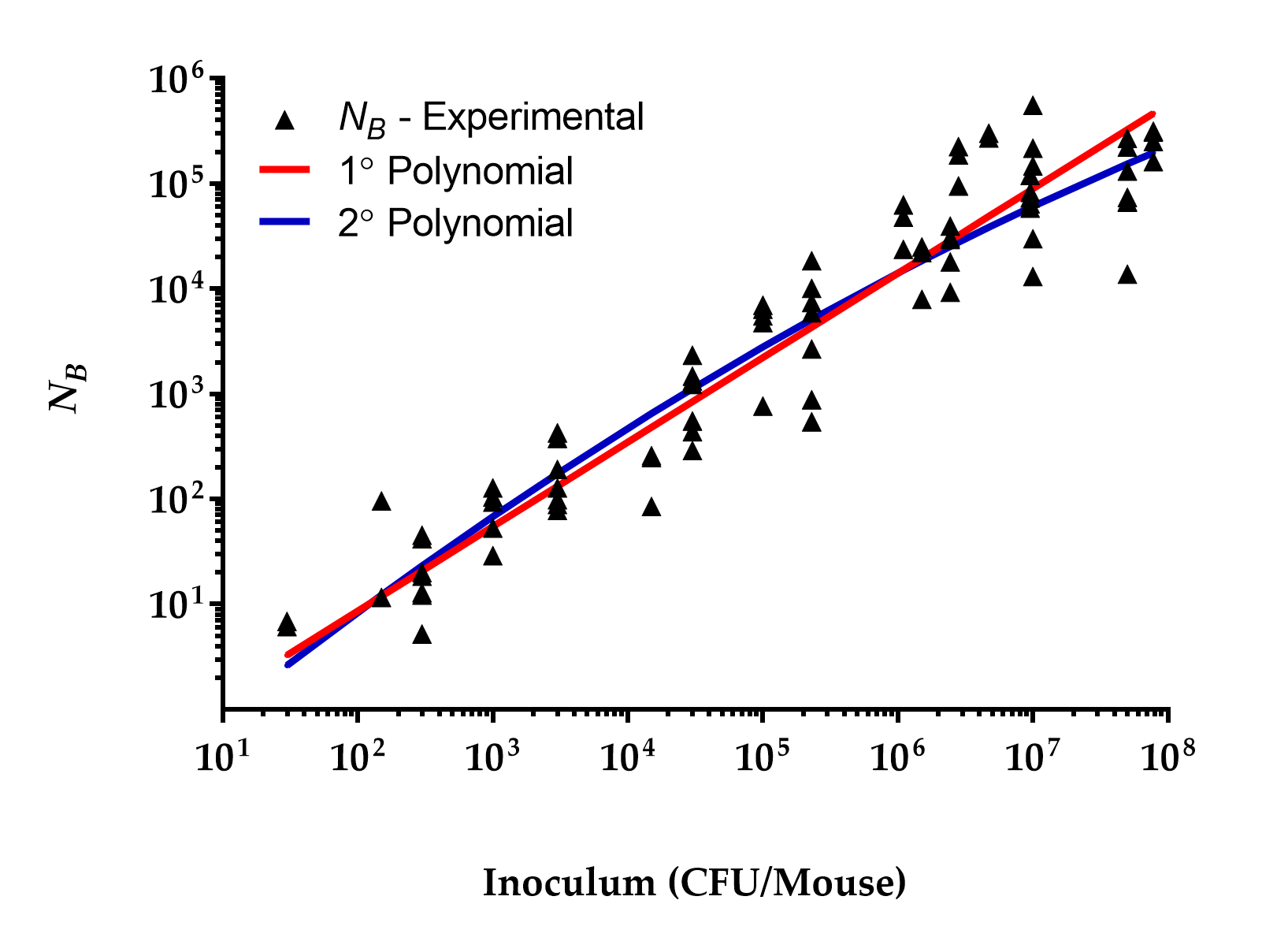

Supplement: FIG S2 [file msystems.00659-21-sf002.tif]

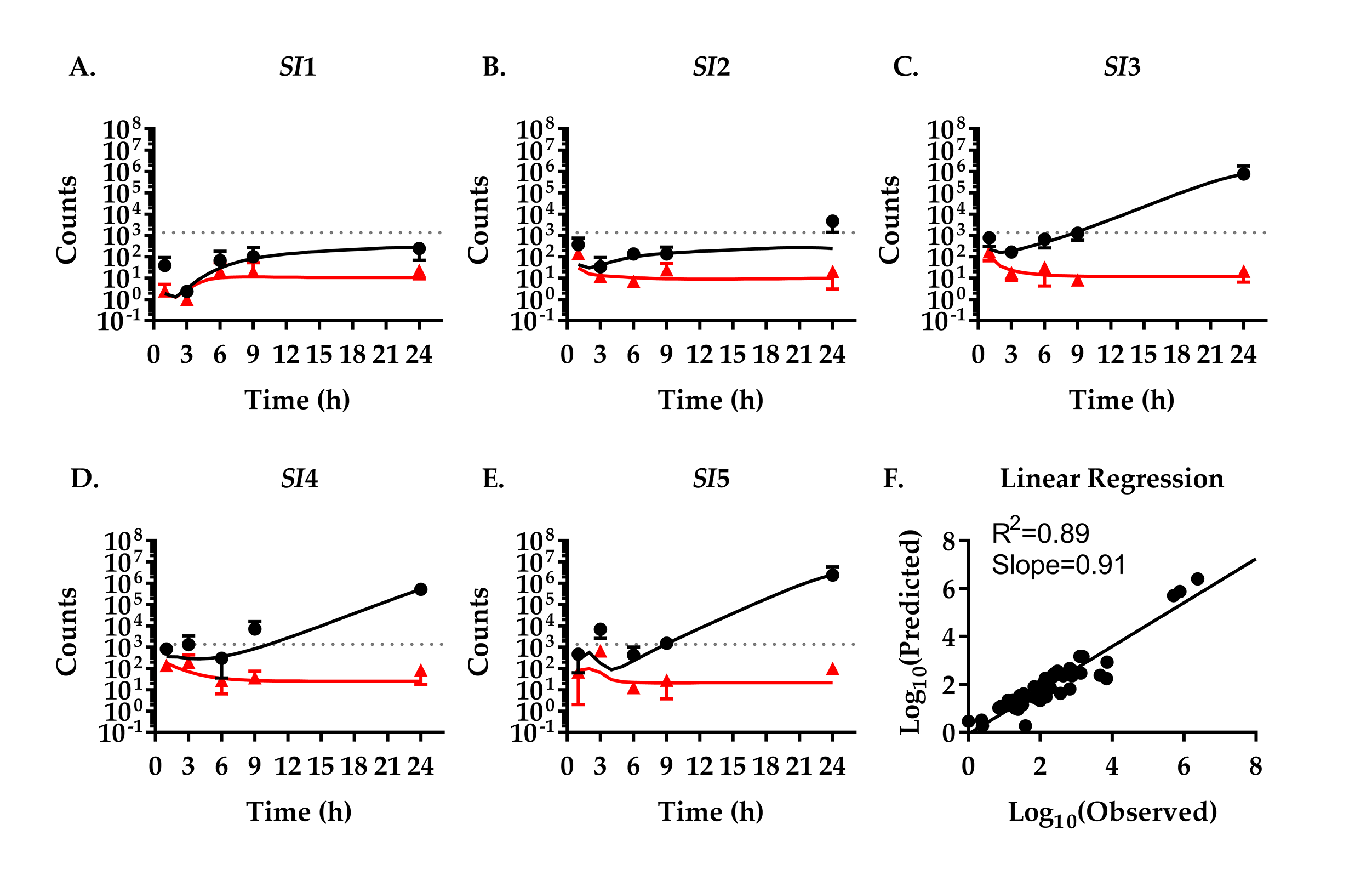

Supplement: FIG S3 [file msystems.00659-21-sf003.tif]

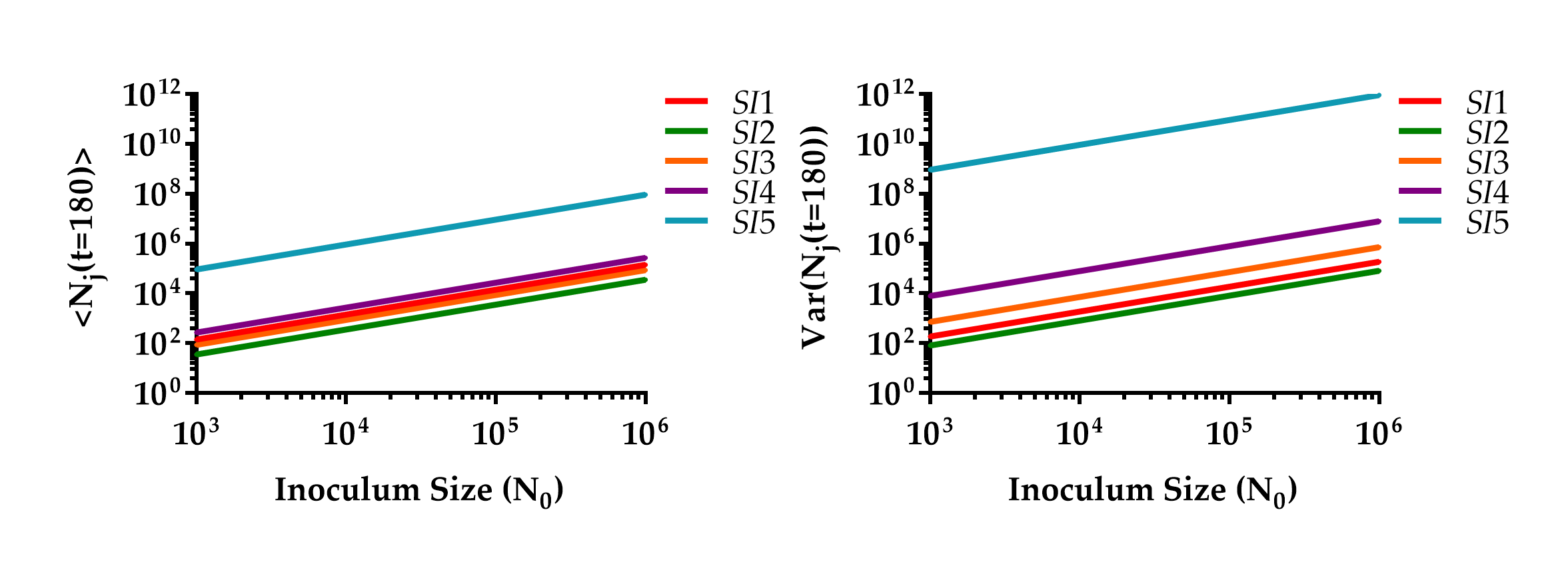

Supplement: FIG S4 [file msystems.00659-21-sf004.tif]

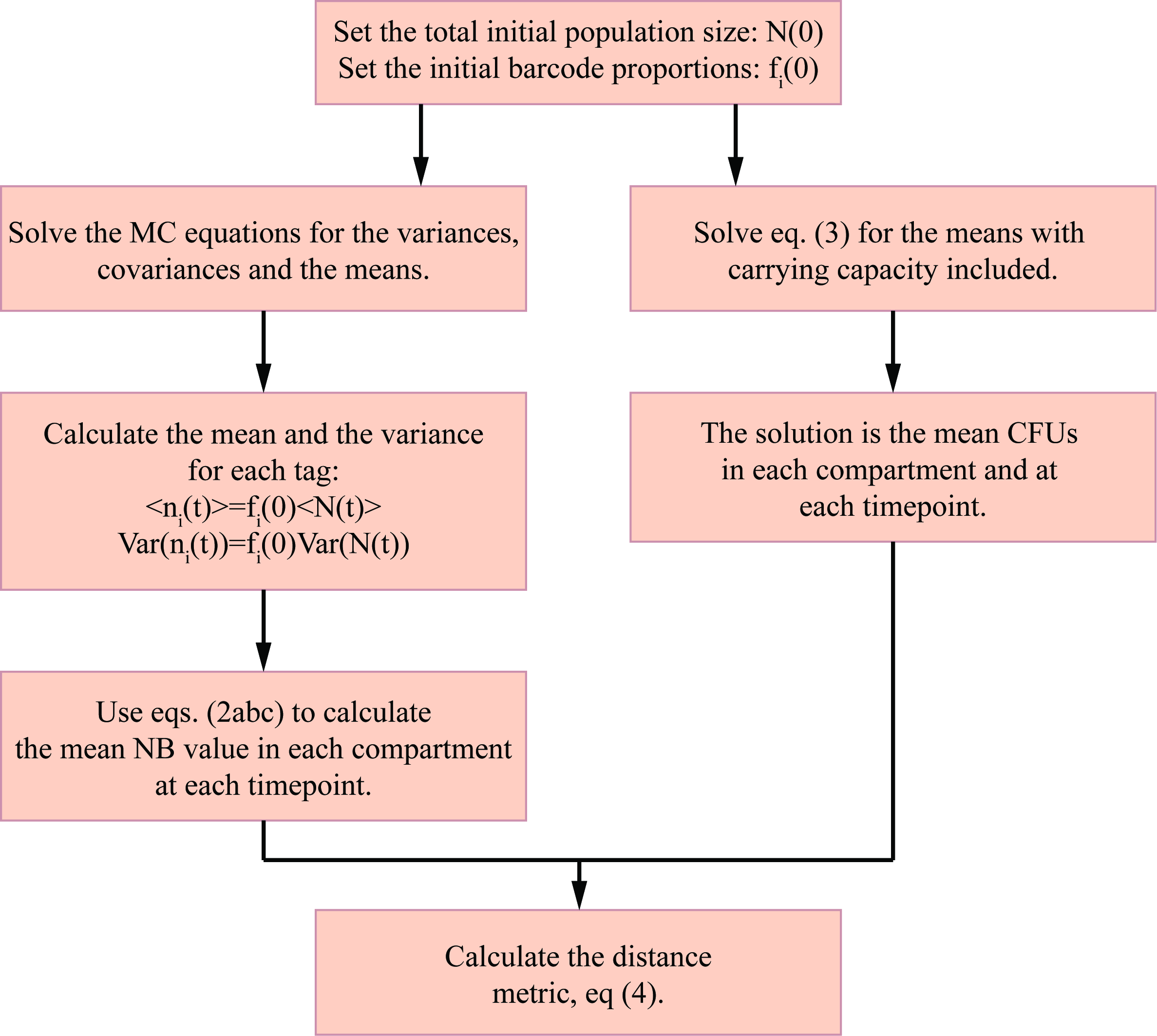

Supplement: FIG S5 [file msystems.00659-21-sf005.tif]

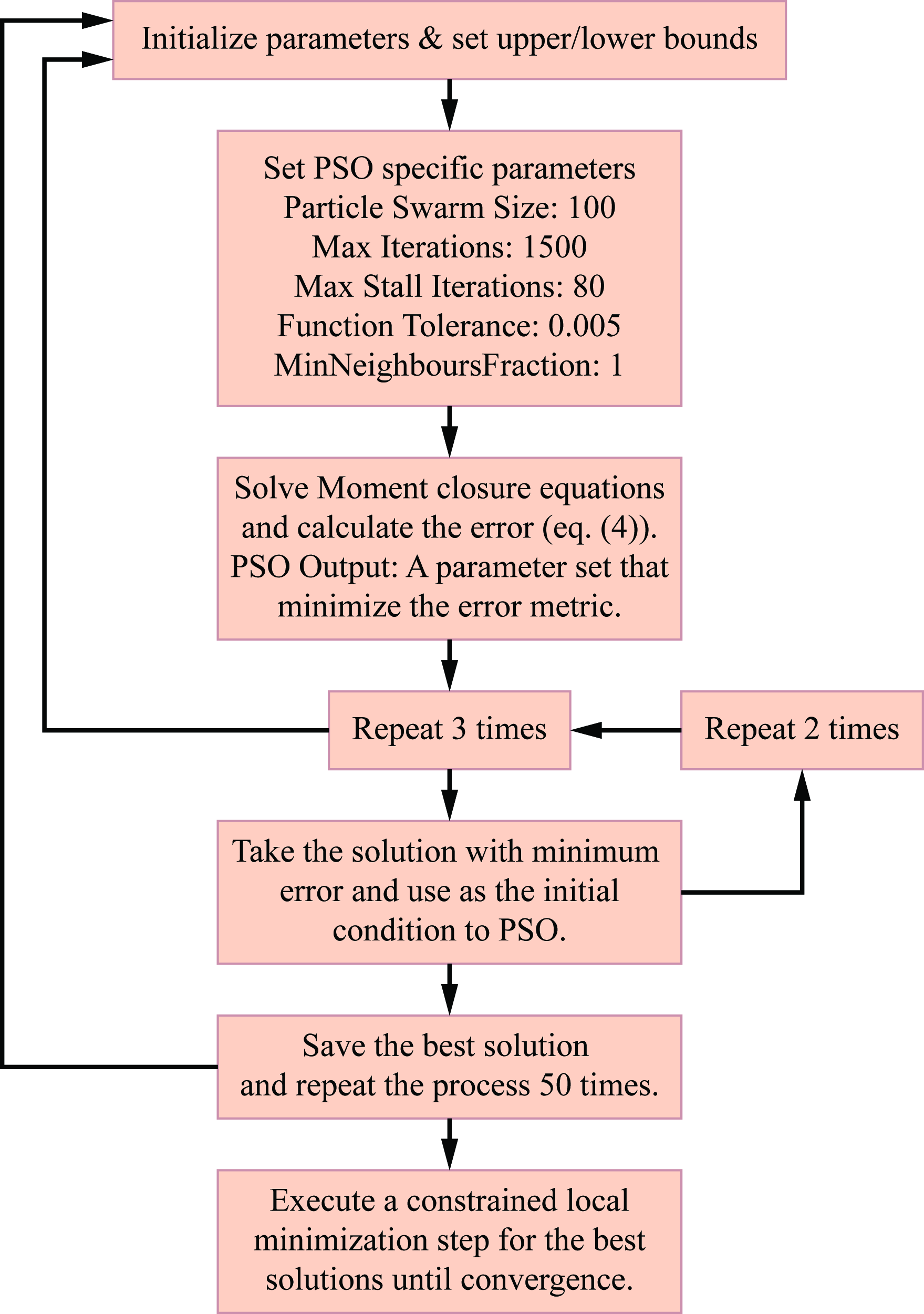

Supplement: FIG S6 [file msystems.00659-21-sf006.tif]
