# Supplementary material for: The Infectious Dose Shapes Vibrio cholerae Within-Host Dynamics
Source: mSystems. 2021 Dec 7;6(6):e00659-21. doi: 10.1128/mSystems.00659-21 (PMC8651084; doi:10.1128/mSystems.00659-21)
Supplement: TEXT S1 [file msystems.00659-21-s0001.docx]

**Supplementary Material**

**Text S1. Relating the founder population size to the mean and the variance of the CFUs.** We derive the relationship between the mean founder population size, the mean CFU and the variance of the CFU by first recognizing that the proportion of subpopulation *i* at time t, *f_i_(t)*, is a random variable and changes by means of random birth-death events. Consequently, the founder population size given by equation (1) is also a random variable. We suppress subscript *j* to minimize notational cluttering and proceed by taking the mean on both sides of eq. (1) which gives,

$<NB^{-1}>=\frac{1}{k}\sum_{i=1}^{k} \frac{<(f_{i}(t)-f_{i}(0))^{2}>}{f_{i}(0)(1-\mathrm{f}_{i}(0))}=\frac{1}{k}\sum_{i=1}^{k} \frac{<NB_{i}^{-1}>}{f_{i}(0)(1-\mathrm{f}_{i}(0))}$ (1s)

where we introduce the auxiliary variable

$<NB_{i}^{-1}>=<(f_{i}(\mathrm{t})-\mathrm{f}_{i}(0))^{2}>$. (2s)

Expanding the square in equation 2s and the proportions of subpopulations at time t in terms of cell numbers we get,

$<NB_{i}^{-1}>=<\frac{n_{i}(t)^{2}}{N(t)^{2}}>-2f_{i}(0)<\frac{n_{i}(t)}{N(t)}>+\mathrm{f}_{i}(0)^{2}$ (3s)

Next we use the error propagation technique to estimate the mean of the ratios of the subpopulation size, *n_i_(t)*, and the total population size *N(t)*. For the sake of notational simplicity, we substitute x=*n_i_(t)* and y=*N(t)*. Let,

$\begin{matrix} f(x,y)=\frac{x^{2}}{y^{2}},\frac{\partial^{2}f}{\partial x^{2}}=\frac{2}{y^{2}},\frac{\partial^{2}f}{\partial y^{2}}=\frac{6x^{2}}{y^{4}},\frac{\partial^{2}f}{\partial x\partial y}=-\frac{4x}{y^{3}}=\frac{\partial^{2}f}{\partial y\partial x} \\ g(x,y)=\frac{x}{y},\frac{\partial^{2}g}{\partial y^{2}}=\frac{2x}{y^{3}},\frac{\partial^{2}g}{\partial x\partial y}=-\frac{1}{y^{2}}=\frac{\partial^{2}g}{\partial y\partial x} \end{matrix}$ (4s)

Taylor expanding f(x,y) and g(x,y) about <x>=µ_x_ and <y>=µ_y_ up to second order we get,

$\begin{matrix} f(x,y)\approx f(\mu_{x},\mu_{y})+\left.\frac{\partial f}{\partial x}\right|_{x=\mu_{x,}y=\mu_{y}}(x-\mu_{x})+\left.\frac{\partial f}{\partial y}\right|_{x=\mu_{x,}y=\mu_{y}}(\mathrm{y}-\mu_{y})+\frac{1}{2}\left.\frac{\partial^{2}f}{\partial x^{2}}\right|_{x=\mu_{x,}y=\mu_{y}}(x-\mu_{x})^{2}+\frac{1}{2}\left.\frac{\partial^{2}f}{\partial y^{2}}\right|_{x=\mu_{x,}y=\mu_{y}}(\mathrm{y}-\mu_{y})^{2}+\left.\frac{\partial^{2}f}{\partial x\partial y}\right|_{x=\mu_{x,}y=\mu_{y}}(\mathrm{x}-\mu_{x})(\mathrm{y}-\mu_{y}) \\ \mathrm{g}(x,y)\approx g(\mu_{x},\mu_{y})+\left.\frac{\partial g}{\partial x}\right|_{x=\mu_{x,}y=\mu_{y}}(x-\mu_{x})+\left.\frac{\partial g}{\partial y}\right|_{x=\mu_{x,}y=\mu_{y}}(\mathrm{y}-\mu_{y})+\frac{1}{2}\left.\frac{\partial^{2}g}{\partial x^{2}}\right|_{x=\mu_{x,}y=\mu_{y}}(x-\mu_{x})^{2}+\frac{1}{2}\left.\frac{\partial^{2}g}{\partial y^{2}}\right|_{x=\mu_{x,}y=\mu_{y}}(\mathrm{y}-\mu_{y})^{2}+\left.\frac{\partial^{2}g}{\partial x\partial y}\right|_{x=\mu_{x,}y=\mu_{y}}(\mathrm{x}-\mu_{x})(\mathrm{y}-\mu_{y}) \end{matrix}$(5s)

After taking the mean on both sides, equation 5s reduces to

$\begin{matrix} <f(x,y)>\approx f(\mu_{x},\mu_{y})+\frac{1}{2}\left.\frac{\partial^{2}f}{\partial x^{2}}\right|_{x=\mu_{x,}y=\mu_{y}}Var(x)+\frac{1}{2}\left.\frac{\partial^{2}f}{\partial y^{2}}\right|_{x=\mu_{x,}y=\mu_{y}}Var(y)+\left.\frac{\partial^{2}f}{\partial x\partial y}\right|_{x=\mu_{x,}y=\mu_{y}}Cov(x,y) \\ <\mathrm{g}(x,y)>\approx g(\mu_{x},\mu_{y})+\frac{1}{2}\left.\frac{\partial^{2}g}{\partial x^{2}}\right|_{x=\mu_{x,}y=\mu_{y}}Var(x)+\frac{1}{2}\left.\frac{\partial^{2}g}{\partial y^{2}}\right|_{x=\mu_{x,}y=\mu_{y}}Var(y)+\left.\frac{\partial^{2}g}{\partial x\partial y}\right|_{x=\mu_{x,}y=\mu_{y}}Cov(x,y) \end{matrix}$ (6s)

Before proceeding further, we substitute back x=*n_i_(t)* and y=*N(t)* to resolve the covariance term Cov(*n_i_(t)*,*N(t)*). For independent subpopulations, the covariance term reduces to

$Cov(n_{i}(t),N(t))=Cov(n_{i}(t),\sum_{i=1}^{k} n_{i}(t))=Cov(n_{i}(t),n_{i}(t))=Var(n_{i}(t))$ (7s)

Hence Cov(x,y)=Var(x). Substituting in the partial derivatives in eqs. (4s) and the covariance term (eq. (7s)) in eqs. (6s) we get,

$\begin{matrix} <f(x,y)>\approx\frac{\mu_{x}^{2}}{\mu_{y}^{2}}+\frac{Var(x)}{\mu_{y}^{2}}(1-4\frac{\mu_{x}}{\mu_{y}})+\frac{3\mu_{x}^{2}}{\mu_{y}^{4}}Var(y) \\ <\mathrm{g}(x,y)>\approx\frac{\mu_{x}}{\mu_{y}}+\frac{\mu_{x}}{\mu_{y}^{3}}Var(y)-\frac{1}{\mu_{y}^{2}}Var(x) \end{matrix}$ (8s)

The auxiliary variable, <*N*_B_*_i_^-1^*>, in terms of f(x,y) and g(x,y) is

$<NB_{i}^{-1}>=<f(x,y)>-2f_{i}(0)<g(x,y)>+f_{i}(0)^{2}$ (9s)

Substituting eqs. (8s) in (9s) we get an expression for the auxiliary variable in terms of the mean and the variances of the population sizes,

$<NB_{i}^{-1}>=\frac{\mu_{x}}{\mu_{y}}(\frac{\mu_{x}}{\mu_{y}}-2f_{i}(0))+\frac{Var(x)}{\mu_{y}^{2}}(1-4\frac{\mu_{x}}{\mu_{y}}+2f_{i}(0))+\frac{\mu_{x}}{\mu_{y}^{3}}Var(y)(3\frac{\mu_{x}}{\mu_{y}}-2f_{i}(0))+\mathrm{f}_{i}(0)^{2}$ (10s)

Equations 1s and 10s correspond to equations 2ab after substituting the standard variable notation x, y, µ_x_ and µ_y_ for the population sizes whereas equation 2c is a first order approximation of the mean founder population size in compartment *j*, <*N*_B_*_j_*>, as 1/<*N*_B_*_j_^-1^*>.
